# Case report: Toxic epidermal necrolysis induced by tislelizumab in a patient with esophageal squamous cell carcinoma

**DOI:** 10.3389/fmed.2024.1522525

**Published:** 2024-12-23

**Authors:** Shilong Wu, Chenyang Xu, Huafeng Liu

**Affiliations:** ^1^Department of Thoracic Surgery, Ganzhou People’s Hospital, Ganzhou, China; ^2^Department of Oncology, Ganzhou People’s Hospital, Ganzhou, China

**Keywords:** toxic epidermal necrolysis, tislelizumab, esophageal squamous cell carcinoma, immune-related adverse events, skin toxicity

## Abstract

**Background:**

Immune checkpoint inhibitors (ICIs) have been widely applicated for the treatment of patients with advanced esophageal cancer. Skin-related adverse reactions are frequent with ICIs, with toxic epidermal necrolysis (TEN) being a severe and potentially life-threatening cutaneous reaction.

**Case presentation:**

We present a case of a 70-year-old male with locally advanced esophageal cancer who developed severe toxic epidermal necrolysis (TEN) after 18 days of tislelizumab combined with chemotherapy. The condition rapidly progressed to cover approximately 90% of his body. After treatment with intravenous methylprednisolone, immunoglobulin, and antibiotics, along with active nutritional support and wound care, the patient recovered from TEN induced by tislelizumab.

**Conclusion:**

Treatment for TEN is complex, and no standardized guidelines currently exist. We propose an economical, safe, effective, and simple strategy for similar TEN patients.

## 1 Introduction

The widespread use of immune checkpoint inhibitors (ICIs) for treating esophageal squamous cell carcinoma (ESCC) has drawn attention to immune-related adverse events (ir-AEs) (1, 2). Skin toxicity is one of the most prevalent adverse reactions for ICIs (3). Toxic epidermal necrolysis (TEN) is a rare but potentially fatal reaction characterized by sudden widespread erythema and skin peeling, often accompanied by mucositis and fever, affecting more than 30% of the body surface area (4). Here, we presented a case of severe TEN that was induced by tislelizumab in a patient with esophageal squamous cell carcinoma.

## 2 Case report

**Figure 1:**
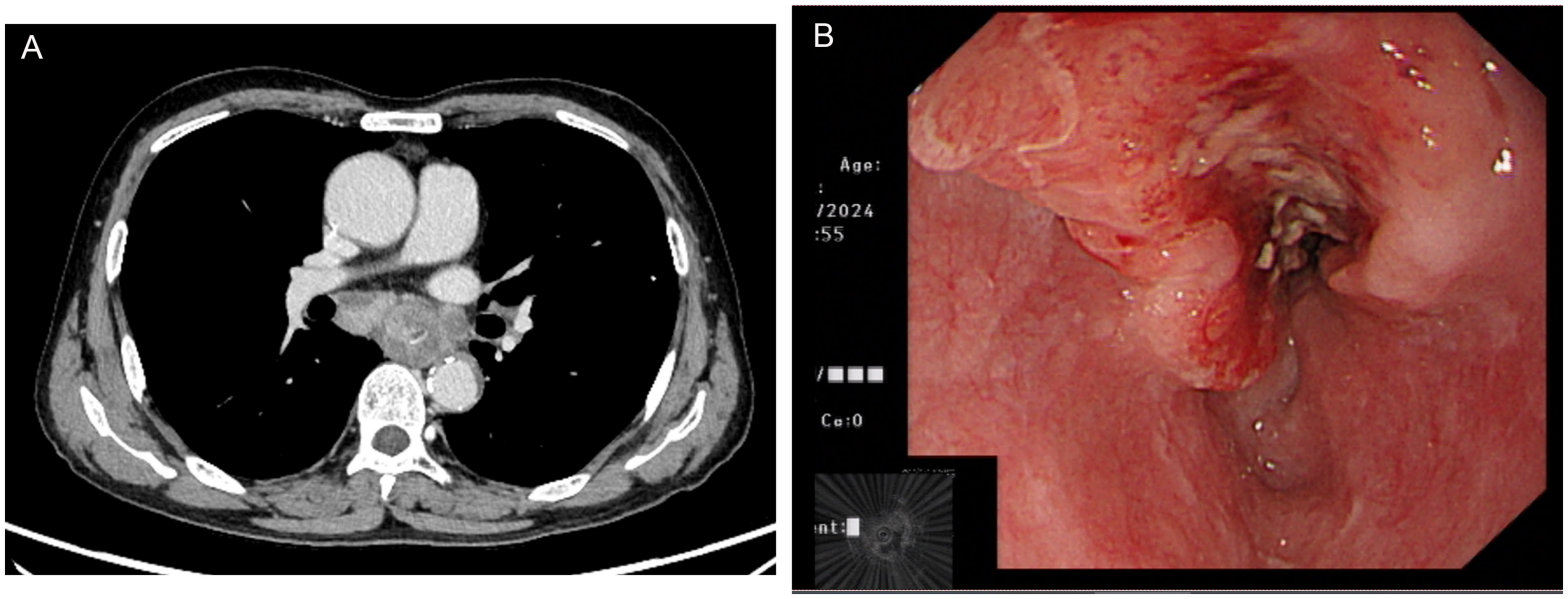
Chest computed tomography **(A)** and endoscopic ultrasonography **(B)** showed esophageal squamous cell carcinoma in the mid-chest area.

**Figure 2:**
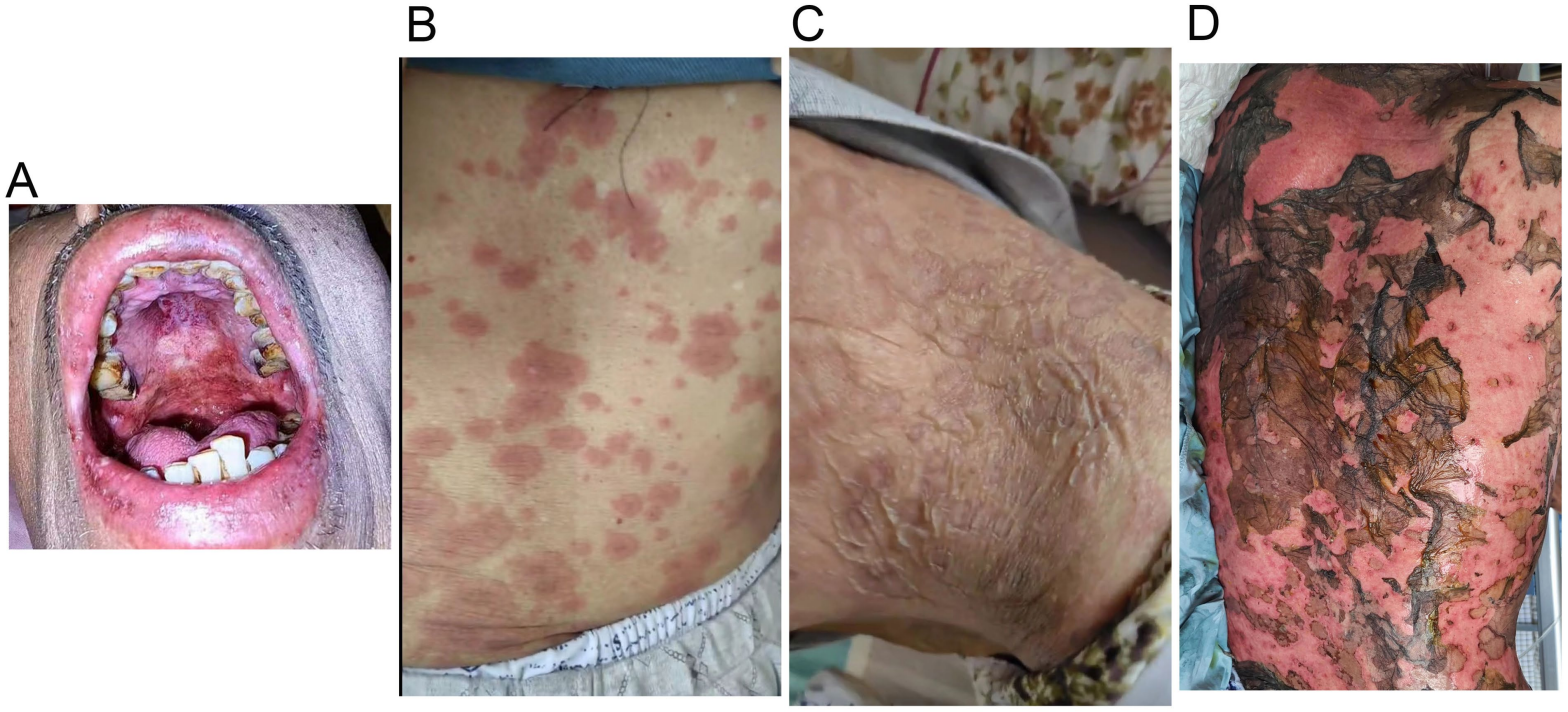
Tislelizumab-related skin toxicity. **(A)** Oral mucositis; **(B)** Rashes of the trunk; **(C)** Blisters; **(D)** Toxic epidermal necrolysis.

**Figure 3:**
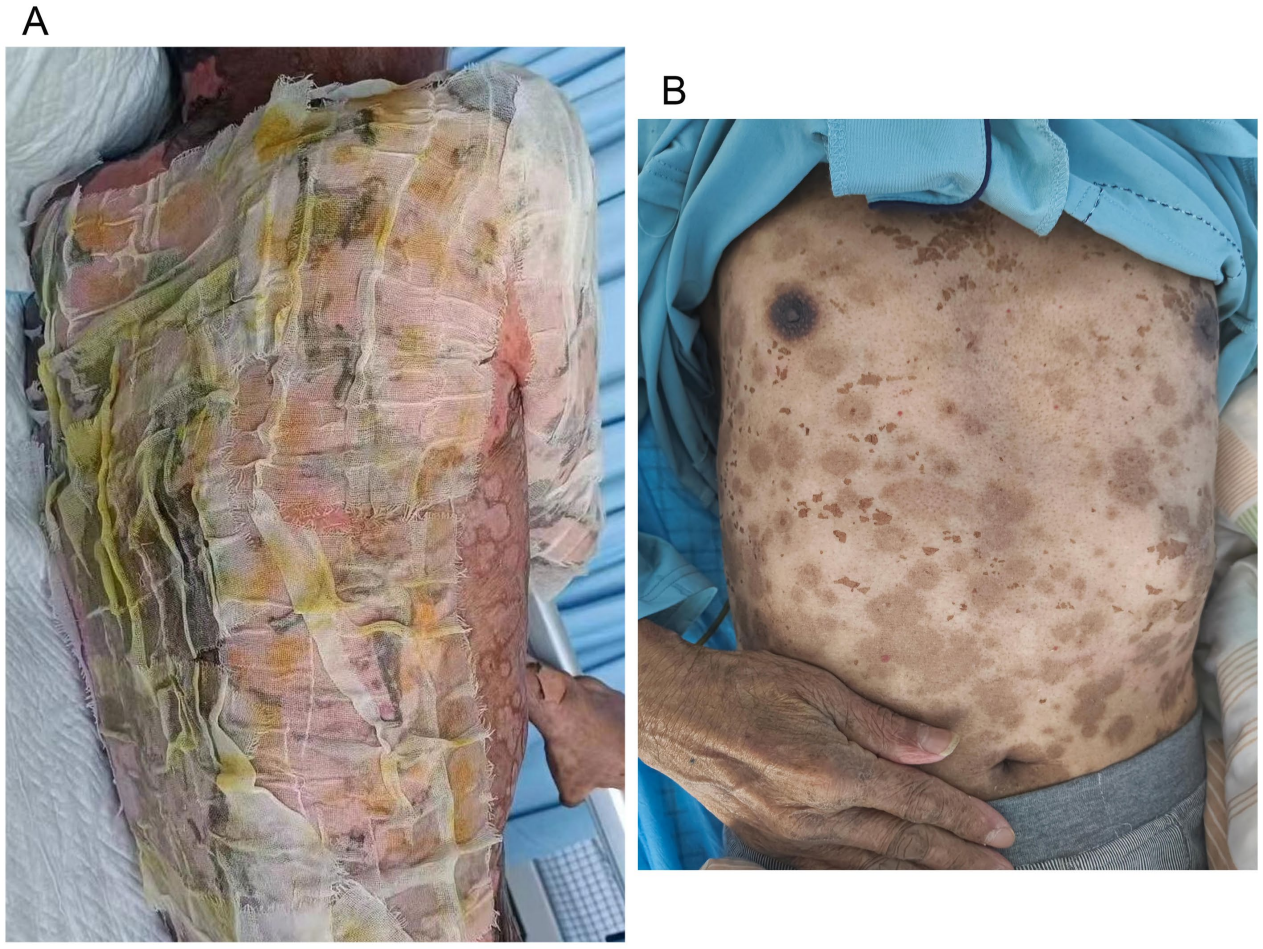
Wound care for toxic epidermal necrolysis **(A)**. An image of the skin after recovery from toxic epidermal necrolysis **(B)**.

**Figure 4:**
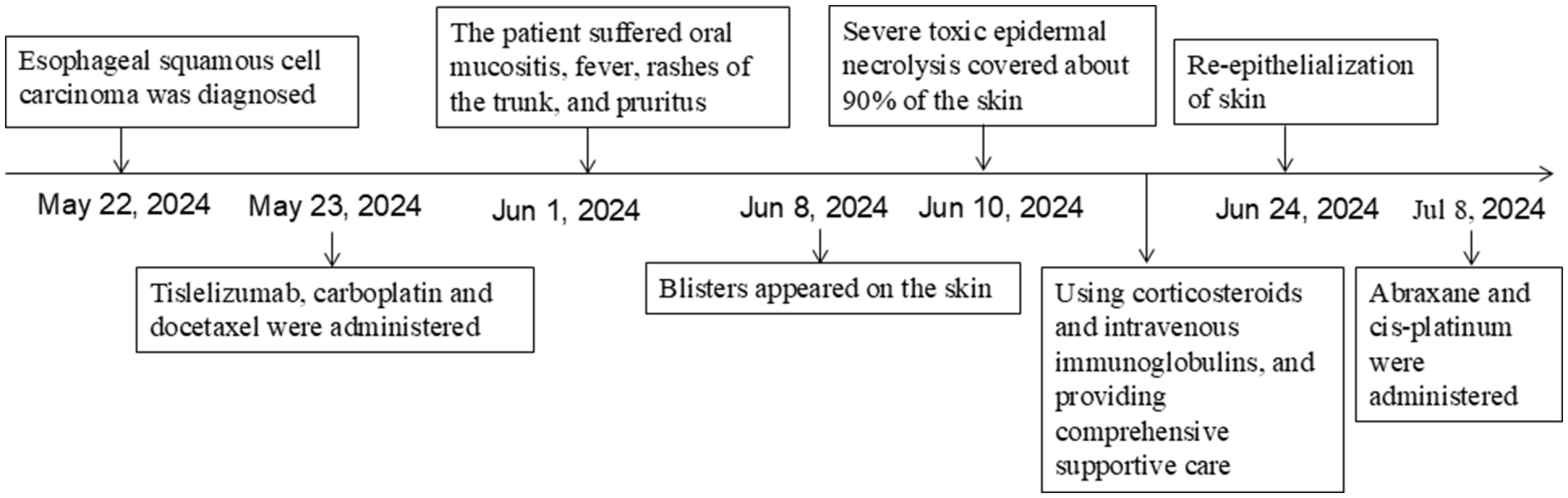
Schematic representation of the patient’s treatment history.

A 70-year-old man was admitted to our hospital due to dysphagia. The patient had coronary stenting 2 weeks prior. The chest computed tomography scan and endoscopic ultrasonography revealed ESCC (Supplementary file 1) in the mid-chest area (Figure 1). For the patient’s locally advanced ESCC, neoadjuvant chemoradiotherapy or neoadjuvant immunotherapy plus chemotherapy was recommended. Tislelizumab (200 mg), carboplatin (450 mg) and docetaxel (110 mg) were administered on May 23, 2024. Nine days post-therapy, the patient suffered oral mucositis (Figure 2A), fever, rashes of the trunk (Figure 2B), and pruritus. Patient received oral prednisone (20–40 mg/d), loratadine (10 mg/d), levocetirizine (5 mg/d), and levofloxacin (500 mg/d). Skin toxicity still continued to progress, resulting in numerous blisters (Figure 2C) on the trunk after 16 days. After 18 days, the patient was admitted to our hospital due to the progression of skin toxicity to TEN (Figure 2D). The cutaneous lesions covered about 90% of the body’s surface. Following admission, urgent consultations were requested from the dermatology and oncology departments. Skin biopsies revealed subepidermal blisters with necrosis, apoptotic keratinocytes, and a lymphocytic inflammatory infiltrate (Supplementary file 2). The SCORTEN score (5) for severity assessment was calculated at 3 (including age above 40 y, malignancy and initial percentage of epidermal detachment above 10%). The ALDEN algorithm was utilized to evaluate drug causality in epidermal necrolysis (6). Tislelizumab had an ALDEN score of 6, and docetaxel’s score was 4. The patient was immediately given prophylactic intravenous antibiotics, intravenous methylprednisolone (2–3 mg/kg/day for 3 days) and intravenous immunoglobulin (400 mg/kg/day for 3 days). Active nutritional support and electrolyte balance were maintained simultaneously. After applying mupirocin ointment and recombinant bovine basic fibroblast growth factor gel to the limbs and trunk, cover with vaseline sand blocks (Figure 3A). Daily disinfect the skin using iodophor, aspirate the blister with a syringe, and softly debride the large detached epidermal segments. Recombinant bovine basic fibroblast growth factor eye drops and levofloxacin eye drops were applied to the eyes. Methylprednisolone was gradually reduced and discontinued after 11 days as symptoms improved. No skin infection developed during treatment, and re-epithelialization occurred two weeks after admission (Figure 3B). However, fever developed due to infection of the subclavian vein catheter. *Escherichia coli* was isolated from the vein catheter. The body temperature gradually normalized following the administration of sensitive antibiotics (ceftazidime). Abraxane (380 mg) and cis-platinum (100 mg) were administered on July 8, 2024. Figure 4 presented the patient’s treatment timeline. No new skin toxicity developed after the second chemotherapy. Radiotherapy (PTV 60GY/30F) was given on August 30, 2024. The patient was lost to follow-up after radiotherapy.

## 3 Discussion

Immune checkpoint inhibitor therapy has now become the standard treatment for advanced ESCC. Recent trials have indicated that the use of ICI combination chemotherapy in the perioperative settings may enable a high PCR rate, complete resection rate, acceptable safety profiles, and thus lead to better survival outcomes (2, 7). Although the safety of ICIs was manageable in most studies, severe cardiovascular, respiratory, endocrine disorders and severe skin toxicity have been reported (2). To the best of our knowledge, this is the first report of TEN induced by tislelizumab in a patient with ESCC.

A recent meta-analysis found that ICIs significantly increased the rates of ir-AEs in esophageal or gastroesophageal junction cancer, but did not significantly raise the rates of serious treatment-related adverse events (TRAEs). The most common irAEs were skin reaction, with varying toxicity manifestations among different ICIs (8). As we know, no study has reported TEN induced by tislelizumab in a patient with ESCC. Among single ICI therapies, sintilimab had the highest incidence of TEN (9). The RATIONALE-306 study showed that 97% of patients in the tislelizumab plus chemotherapy group experienced TRAEs, with myelosuppression being the most common grade 3 or 4 TRAE. The study indicated that skin toxicity with stomatitis and pruritis, but without the occurrence of TEN (10).

Stevens–Johnson syndrome (SJS) and TEN is a type IV hypersensitivity reaction triggered by an immunologic response, most often to drugs (11). SJS/TEN occurs in 1–5 cases per million individuals annually, with a higher incidence in adults than in pediatric patients (12). Identifying the drug causing SJS/TEN is essential, and ALDEN is a valuable tool for assessing drug causality in this condition (6). Compared to Non-ICI drugs, patients with ICI-induced SJS/TEN had a smaller affected body surface area, a trend of prolonged latency, and milder oral and ocular mucositis (13). SJS/TEN usually occurs early in treatment, with a median onset of 23 days after starting ICI, typically following 1–2 cycles (14). We could not definitively determine if immunotherapy or chemotherapy led to TEN before the second round of chemotherapy. The ALDEN score was highest with tislelizumab and decreased to 2 with docetaxel after the second round of chemotherapy. So we concluded that tislelizumab caused TEN. The patient developed TEN on the 18th day following the initial immunotherapy in our case. The overall mortality rate of TEN was 32%, primarily due to infections and tumor progression, with a median interval of 28 days from SJS/TEN onset to death. Deceased patients had a notably higher proportion of TEN and a greater epidermal detachment area than survivors (14). TEN leads to the permanent withdrawal of immunotherapy and the delay of anti-tumor therapy in this patient.

There are no standardized guidelines for treatment SJS/TEN, some organizations recommend permanently discontinuing ICIs, using corticosteroids and intravenous immunoglobulins, and providing comprehensive supportive care (15, 16). Given the rapid progression of SJS/TEN, with peak skin detachment typically occurring within 8 days, prompt initiation of corticosteroids is essential due to the prognostic significance of skin loss extent (17). Methylprednisolone was administered at an elevated dose due to the rapid progression of TEN in our case. The extent of epidermal detachment is an important prognostic factor in ICI-related cases, likely due to the heightened risk of infection and metabolic disturbances linked to extensive skin involvement (14). There is currently no consensus on optimal wound care. Gentle debridement of large detached epidermal segments, aspiration of bulla fluid, and anti-shear measures, along with adjunct immunomodulatory therapies, is the preferred approach for most cases (18). A recent systematic review showed that patients with ICI-related SJS/TEN typically experienced re-epithelization within 30 days. In our case, re-epithelization occurred in 14 days, presenting a simple and effective wound care method. What’s more, managing intravenous transfusions was challenging.

## 4 Conclusion

In conclusion, tislelizumab-related TEN in esophageal cancer treatment is a very rare but serious adverse event. It is crucial to monitor skin reactions during and after treatment with anti-PD-1 agents. Active systemic therapy, supportive treatment, and wound care are crucial. We present a cost-effective, safe, efficient, and simple strategy that we recommend for similar TEN patients. However, the limited number of cases prevents us from verifying the strategy’s universality.

## Data Availability

The original contributions presented in the study are included in the article/Supplementary material, further inquiries can be directed to the corresponding author.
