# Supplementary figures and images for: Case report: Toxic epidermal necrolysis induced by tislelizumab in a patient with esophageal squamous cell carcinoma
Source: Front Med (Lausanne). 2024 Dec 23;11:1522525. doi: 10.3389/fmed.2024.1522525 (PMC11700972; doi:10.3389/fmed.2024.1522525)

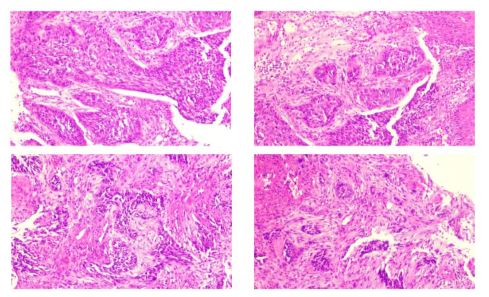

Supplement: Supplementary file 1 [file Image_1.JPEG]

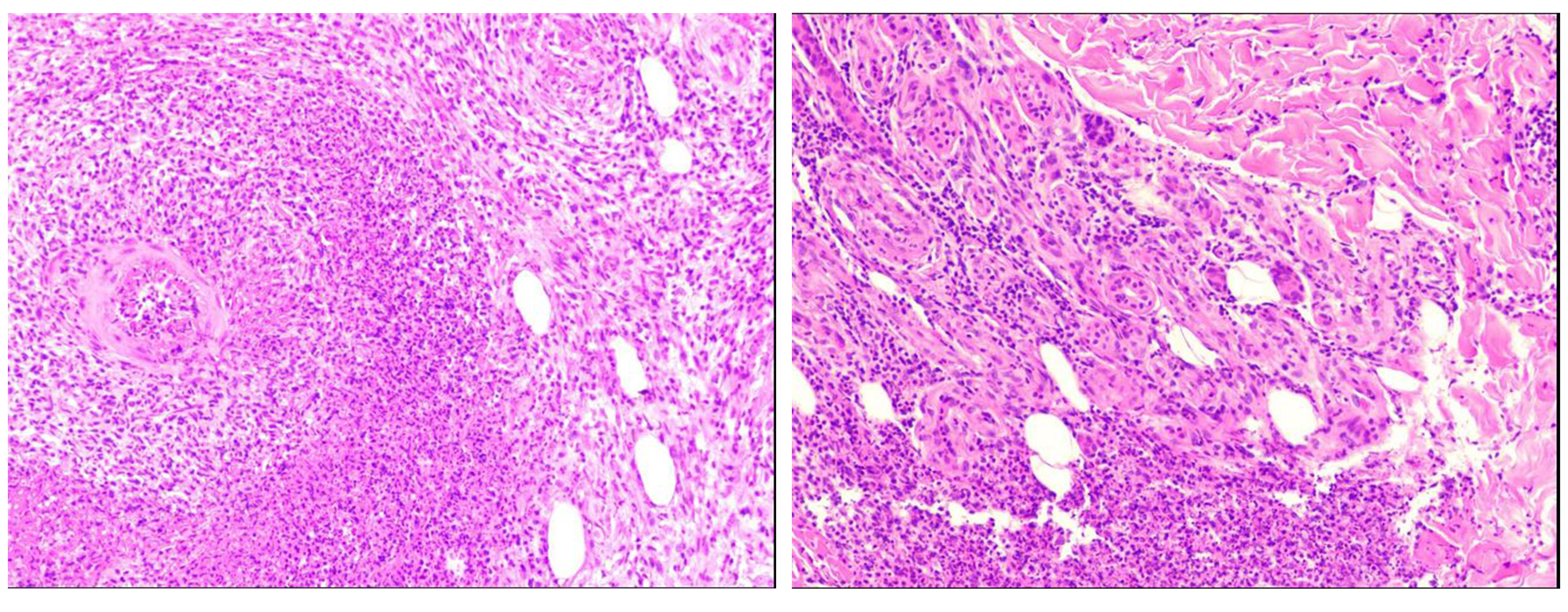

Supplement: Supplementary file 2 [file Image_2.JPEG]
